# Formulation and Characterization of Native and Crosslinked Hyaluronic Acid Microspheres for Dermal Delivery of Sodium Ascorbyl Phosphate: A Comparative Study

**DOI:** 10.3390/pharmaceutics10040254

**Published:** 2018-12-01

**Authors:** Arianna Fallacara, Filippo Marchetti, Michele Pozzoli, Ugo Raffaello Citernesi, Stefano Manfredini, Silvia Vertuani

**Affiliations:** 1 Department of Life Sciences and Biotechnology, Master Course in Cosmetic Science and Technology (COSMAST), University of Ferrara, Via Luigi Borsari 46, 44121 Ferrara (FE), Italy; fllrnn@unife.it (A.F.); filippo.marchetti@student.unife.it (F.M.); vrs@unife.it (S.V.); 2 Respiratory Technology, Woolcock Institute of Medical Research and Discipline of Pharmacology, Faculty of Medicine and Health, The University of Sydney, 431 Glebe Point Road, Glebe, NSW 2037, Australia; michele.pozzoli@sydney.edu.au; 3 I.R.A. Istituto Ricerche Applicate s.r.l., Via Del Lavoro 4a/6, 20865 Usmate-Velate (MB), Italy; citernesi@iralab.it; 4 Ambrosialab Srl, Via Mortara 171, 44121 Ferrara (FE), Italy

**Keywords:** dermal delivery, drug release, hyaluronic acid, urea-crosslinked hyaluronic acid, microspheres, sodium ascorbyl phosphate

## Abstract

The present work evaluates for the first time the use of urea-crosslinked hyaluronic acid (HA-CL), a novel derivative of native hyaluronic acid (HA), to produce microspheres (MS) by emulsification-solvent evaporation, for dermal delivery of sodium ascorbyl phosphate (SAP). As the term of comparison, HA MS were prepared. A pre-formulation study—investigation of the effects of polymers solutions properties (pH, viscosity) and working conditions—led to the production of optimized HA-CL MS and HA-CL—SAP MS with: almost unimodal size distributions; mean diameter of 13.0 ± 0.7 and 9.9 ± 0.8 µm, respectively; spherical shape and rough surface; high yield, similar to HA MS and HA–SAP MS (≈ 85%). SAP was more efficiently encapsulated into HA-CL MS (78.8 ± 2.6%) compared to HA MS (69.7 ± 4.6%). Physical state, thermal properties, relative moisture stability of HA-CL MS and HA-CL–SAP MS were comparable to those of HA MS and HA–SAP MS. However, HA-CL–SAP MS exhibited an extended drug release compared to HA–SAP MS, despite the same kinetic mechanism—contemporaneous drug diffusion and polymer swelling/dissolution. Therefore, HA-CL formulation showed a greater potential as microcarrier (for encapsulation efficiency and release kinetic), that could be improved, in future, using suitable excipients.

## 1. Introduction

Hyaluronic acid (HA) is a naturally occurring glycosaminoglycan, pervasively diffused in the human body: it is found in the extracellular matrix, skin dermis, eye vitreous, hyaline cartilage, synovial fluid, and umbilical cord. HA is well known for its numerous biological functions [1,2] and its interesting properties such as biocompatibility, biodegradability, and mucoadhesion [3,4]. Moreover, HA has moisturizing, lubricant, and filler actions, and it is involved in wound healing and anti-inflammatory processes [1,3]. For these reasons, hyaluronan has widespread applications in medical, pharmaceutical, and cosmetic fields, and represents an interesting starting material—frequently combined to other active ingredients or excipients—in tissue engineering, viscosupplementation, and drug delivery [1,3,4,5,6,7,8,9,10,11,12]. To design biomaterials with improved physical-chemical, viscoelastic, and biological properties, native HA is often subjected to derivatization or crosslinking [13]. Usually, HA is crosslinked with difunctional molecules of synthetic origin, for example, divinyl sulfone and diglycidyl ether [14,15]. Nevertheless, the recent trend consists in crosslinking the polymer with substances characterized by lower toxicity and intrinsic health activity. The aim is to obtain cross-polymers that can act as multifunctional molecules able to deliver active ingredients and to exert, at the same time, a health action [1]. Toward this end, we are actually investigating the possible pharmaceutical, cosmetic, and aesthetic applications of the new HA crosslinked with urea (HA-CL) [9,16,17,18]. HA-CL is a recently patented biocompatible and biodegradable polymer, provided with greater consistency and bioactivity with respect to native HA [9,16,18]. This is due to hyaluronan crosslinking with urea, a molecule naturally present in the human body and also employed as active substance. Indeed, urea is widely and safely used in pharmaceutical and cosmetic formulations because it is keratolytic and moisturizing, and thus it enhances cellular regeneration and repair. Urea is useful to treat different diseases, such as dry skin, damaged cutaneous annexes, non-infectious keratopathy, and injured corneal epithelium [19,20,21]. Hence, HA-CL could be a promising biomaterial for topical treatments requiring simultaneous re-epithelialization and hydration for the resolution of aesthetic/functional skin and mucosae problems [9,16,17]. 

Biodegradable and mucoadhesive polymers, in the form of microparticulate systems such as microspheres (MS), can accelerate skin wound healing [22] and extend the release of the encapsulated drugs [8,23,24]. Acknowledging the aforementioned facts and properties of HA-CL, we decided to explore for the first time, with this work, the potentiality of HA-CL to formulate drug loaded MS intended for the dermal target. The preparative method chosen was a water-in-oil (w/o) emulsification solvent evaporation technique, and native HA was used as reference polymer, as it has been already employed to produce MS [8,11]. This study has as its goal the provision of the perfect case scenario: to have an active molecule, never loaded before into hyaluronan microcarriers, which could be satisfyingly encapsulated and then freely released by the MS. Therefore, sodium ascorbyl phosphate (SAP) was selected as the model drug for its high hydrophilicity [25] and for its unprecedented encapsulation into hyaluronan MS. Moreover, SAP seemed to be an optimal candidate as it is characterized by good physical–chemical stability instead of ascorbic acid, and by several biological activities which go in synergy with those of HA. Indeed, SAP acts as a radical scavenger, with high capacity to reduce damages caused by photo-oxidation and lipid peroxidation, and it has strong antimicrobial activity on *Propionibacterium acnes*, the major bacterium responsible of acne vulgaris [25,26]. The SAP is a non-irritating prodrug bioconverted by skin enzymes into ascorbic acid, which stimulates collagen synthesis and, therefore, increases skin elasticity [27,28]. Hence, the combination of vitamin C derivatives and hyaluronan could open interesting perspectives. In fact, a recent study reported the safety of an HA sponge system containing a derivative of vitamin C used to reduce and treat scars [27]. Additionally, the delivery of a combination of SAP and HA-CL showed enhanced anti-inflammatory and antioxidant activities with respect to the single SAP and HA-CL: hence, the association of SAP and HA-CL could be suitable as an adjunctive therapy for the treatment of inflammatory pulmonary disorders [18].

In this research, SAP-loaded hyaluronan MS were formulated using the novel urea-crosslinked hyaluronic acid. A pre-formulation study was carried out to obtain optimized MS: particle features such as mean diameter, size distribution, yield (Y%), drug loading (DL%), and encapsulation efficiency (EE%) were investigated in relation to the properties of the starting polymeric solutions and to the emulsification time. The optimized MS were then characterized more in detail for their physical–chemical properties—morphology, physical state, thermal behavior, moisture sorption, and stability—and for their in vitro release profiles. An accurate and itemized theoretical study was performed to understand and explain, with a systematic approach, the mechanisms of release and the experimental features of HA-CL formulations. Considering that this was the first research describing HA-CL MS, no excipients were added to the formulations, in order to investigate the actual polymer potentiality as microcarrier. Furthermore, all the properties of SAP-loaded as well as unloaded MS of HA-CL were compared to SAP-loaded and unloaded MS of native HA (prepared as reference formulations). 

## 2. Materials and Methods 

### 2.1. Materials

Native hyaluronic acid (sodium salt, molecular weight 1.2 MDa) and urea-crosslinked hyaluronic acid (molecular weight 2.0–4.0 MDa –raw material containing also pentylene glycol) were kindly given by I.R.A. Srl (Istituto Ricerche Applicate Srl, Usmate-Velate, Monza-Brianza, Italy). Sodium ascorbyl phosphate (known under the trade name STAY-C^®^50) was purchased from DSM Nutritional Products Ltd. (Segrate, Milano, Italy). Phosphate buffered saline (PBS) and hexane were supplied by Sigma-Aldrich (Schnelldorf, Germany). Mineral oil was obtained from Fagron (Quarto Inferiore, Bologna, Italy). Sorbitan monooleate (Span 80) was provided by Acef (Fiorenzuola D’Arda, Piacenza, Italy).

### 2.2. Prepair of Hyaluronan and Hyaluronan-SAP Solutions (Aqueous Phases)

HA 1% (*w*/*v*) solution and HA-CL 1% (*w*/*v*) solution were achieved through a progressive dispersion of the polymers in deionized water, under continuous magnetic stirring (300 rpm). Likewise, hyaluronan-SAP solutions 1% (*w*/*v*), 1:1, were prepared by dispersing the polymers into SAP water solutions, under constant magnetic stirring (300 rpm). The polymers were allowed to completely hydrate thus forming hydrogels, which were left to swell under moderate stirring, over 12 h, at room temperature, to reach homogeneous appearances. The gels were left at rest for 12 h prior to examinations or use as aqueous phases for MS formulation.

### 2.3. Characterization of Hyaluronan and Hyaluronan-SAP Solutions: pH and Rheology

Firstly, the pH of each hyaluronan aqueous phase was measured in triplicate using a digital pH meter (Docu pH+ meter, Sartorius Mechatronics, Goettingen, Germany).

Secondly, hyaluronan hydrogels were subjected to rheological analyses, performed in triplicate, at 23 ± 2 °C, with a rotational rheometer AR2000 (TA Instruments, New Castle, DE, USA), equipped with an aluminum cone/plate geometry—diameter 40 mm, angle 2°, 64 μm truncation. A solvent trap was used in order to prevent samples dehydration. The rheometer was connected to the Rheology Advantage software (version V7.20) for data analysis.

Flow measurements were performed by a shear rate sweep, under steady state condition: after 1-min equilibration time, the shear rate ($\dot{\gamma }$) was progressively increased from 0.01 to 1000 s^−1^. The gels were compared for their zero-shear-rate viscosity (*η*_0_), which was determined by fitting the viscosity curves according to the Cross equation [29] (Equation (1)):

(1) $$\eta =\eta_{\infty }+\frac{\eta_{0}-\eta_{\infty }}{1+{\left(C\cdot \dot{\gamma }\right)}^{n}}$$

where *η* is the viscosity at a given shear rate (Pa.s), $\dot{\gamma }$ is the shear rate (s^−1^), *η_0_* is the zero-shear-rate viscosity (Pa.s), *η*_∞_ is the infinite-shear-rate viscosity (Pa.s), *C* is a multiplicative parameter (s) and *n* is a dimensionless exponent.

Oscillatory measurements were then taken under the constant stress value of 0.2 Pa, which belonged to the viscoelastic linear regime (defined by a strain sweep test), where the hydrogels could not be subjected to irreversible structural modifications. The experiments were carried out with oscillation frequencies ranging from 0.01 to 100 Hz. The elastic modulus (*G*′) and the viscous modulus (*G*″), measured as a function of the frequency of the stress applied, allowed to evaluate the viscoelastic properties of the gels [30]. More precisely, the samples were compared for their elastic modulus at 1 Hz (*G′_1Hz_*, quantitative index of elasticity), and for their crossover frequency (*C_f_*, a frequency where *G*′ is equal to *G*″). 

### 2.4. Formulation of HA and HA-CL Microspheres Containing or not SAP

HA and HA-CL MS containing or not SAP were produced through a water-in-oil (w/o) emulsification solvent evaporation technique, adapted from the method described by Lim and co-workers [11]. 

The aqueous phase was added dropwise (flow rate: 0.91 mL/min) into 100 g of mineral oil (oil phase) containing 1% (*w*/*w*) sorbitan monooleate as the emulsifying agent, under moderate magnetic stirring (200 rpm), at 23 ± 2 °C. The aqueous phase was then emulsified at 1000 rpm, at 23 ± 2 °C, into the oil phase, using a Silverson L5M A Laboratory Mixer (Silverson Machines, Buckinghamshire, United Kingdom), equipped with a fine emulsor screen workhead. Different emulsification times of 10, 30 and 60 min were investigated. Afterward, moderate magnetic stirring (200 rpm) and mild heating (37 ± 2 °C) were constantly maintained for 12 h to guarantee the complete evaporation of the dispersed aqueous phase. The MSs thus formed were separated from the oil phase by centrifugation at 4000 rpm, at 23 ± 2 °C, for 30 min (ALC Centrifuge PK110, OPTO-LAB, Concordia sulla Secchia, Modena, Italy). The pellets were resuspended in hexane and filtered under vacuum, at 23 ± 2 °C, using a Millipore glass filtration system, equipped with a polyamide membrane, pore size 0.22 µm (Sartorius, Muggiò, Monza-Brianza, Italy). The collected MSs were finally dried in an oven at 37 ± 2 °C for 12 h. 

### 2.5. MS Yield, Drug Loading, and Encapsulation Efficiency

MS yield (Y%), drug loading (DL%) and drug encapsulation efficiency (EE%) were respectively calculated from Equations (2)–(4)

(2) $$Y\%=\frac{weight\;of\;recovered\;MS}{weight\;of\;polymer^{*}\;and\;drug\;fed\;initially\;}\cdot 100$$

* HA-CL was provided as a raw material containing pentylene glycol. Being hydrophilic, this excipient was taken into account for the determination of HA-CL MS Y%, because 1% (*w*/*v*) HA-CL solutions contained 0.75% (*w*/*v*) pentylene glycol.

(3) $$DL\%=\frac{weight\;of\;drug\;in\;MS}{weight\;of\;recovered\;MS}\cdot 100$$

(4) $$EE\%=\frac{weight\;of\;drug\;in\;MS}{weight\;of\;drug\;fed\;initially}\cdot 100$$

For each MS formulation, all the determinations were performed in triplicate and the results were reported as the mean ± standard deviation (SD).

The amount of encapsulated drug was determined by completely dissolving 30 mg of SAP loaded MS in 300 mL of release medium. Drug concentration was then assayed by ultraviolet (UV) spectroscopy (SHIMADZU UV-2600 spectrophotometer, Kyoto, Japan), at 258 nm (wavelength value corresponding to SAP λmax), on the basis of a previously plotted calibration curve. Unloaded HA and HA-CL MS were tested to ensure that other components of the formulations were not characterized by UV absorbance at the scanning wavelength.

### 2.6. Particle Size Analysis 

Particle size distributions of HA and HA-CL microspheres containing or not SAP were analyzed using laser diffraction (Malvern Mastersizer 2000, Malvern Instruments Ltd., Malvern, UK). Samples of powder (ca. 10 mg) were dispersed through the Scirocco dry dispersion unit (Malvern, UK) with a feed pressure of 4 bars and a feed rate of 100%. Samples were analyzed in triplicate, with an obscuration value between 0.1% and 15% and a reference refractive index of 1.33. The volume weighted mean diameters (D [4,3]) and the median particle size by volume Dv50 were used to describe MS size. Size distributions were evaluated by calculation of samples *Span* values as (Equation (5))

(5) $$Span=\frac{Dv90-Dv10}{Dv50}$$

where *Dv*90, *Dv*10, and *Dv*50 are respectively the 90%, 10% and 50% cumulative volume distributions. Thus, the *Span* values gave a measure of the ranges of the volume distributions relative to the median diameters.

### 2.7. SEM Morphological Analysis 

The morphology (shape and surface) of HA and HA-CL MS containing or not SAP was observed using a field emission scanning electron microscope (Zeiss EVO 40XVP, Carl Zeiss Pty Ltd., Oberkochen, Germany), with an acceleration voltage of 20 kV. Powder samples were deposited on carbon sticky tabs and sputter coated with a thin layer of gold-palladium, under an argon atmosphere, prior to analysis. The samples were then randomly scanned and photographed. 

### 2.8. X-ray Powder Diffraction

X-ray diffraction measurements on SAP and SAP-loaded and unloaded MS were performed at 40 kV, 40 mA, with the Bruker AXS D8 Advance Geiger counter equipped with a two-dimensional (2D) gas-filled sealed multiwire detector (scattering-angle resolution of 0.02° s^−1^). Monochromatized Cu Kα radiation (λ = 1.54 Å) was used. The analyses were performed in a 5–45° 2ϑ range, at ambient temperature. The intensity vs. scattering angle spectra was obtained after the radial average of the measured 2D isotropic diffraction patterns. Bragg peaks were detected in the wide-angle X-ray diffraction region (WAXD).

### 2.9. Thermal Analysis (DSC and TGA)

The thermal profiles of SAP and MS formulations were studied using differential scanning calorimetry (DSC823e; Mettler-Toledo, Schwerzenbach, Switzerland). Roughly 5 mg of samples were weighted and crimp-sealed in DSC standard 40 µL aluminum pans. Samples were then subjected to a 10 °C/min temperature ramp between −20 °C and 300 °C. The endothermic and exothermic peaks were determined using STARe software V.11.0x (Mettler Toledo, Greifensee, Switzerland). 

Moreover, the temperature stability and solvent evaporation of each sample were determined using thermal gravimetric analysis (TGA; Mettler-Toledo, Schwerzenbach, Switzerland). Approximately 5 mg of samples were placed on aluminum crucible pans. The weight losses of the samples were assessed by heating the samples from 20 °C to 400 °C, with a scanning rate of 5 °C/min, under constant nitrogen gas. Data were analyzed using STARe software V.11.0x (Mettler Toledo, Greifensee, Switzerland) and expressed as the percentage of weight loss comparing to initial sample weight.

### 2.10. Dynamic Vapor Sorption (DVS)

The relative moisture sorption and stability of SAP and MS formulations, with respect to humidity, were analyzed by Dynamic Vapor Sorption (DVS). Aluminum sample pans were loaded with 10 mg ca. of samples and then placed in the sample chamber of a DVS (DVS-1, Surface Measurement Systems Ltd., London, UK). Each sample was dried at 0% relative humidity (RH) before being exposed to 10% RH increments for two 0–90% RH cycles (25 °C). Equilibrium of moisture sorption was determined, at each humidity step, by a change in mass to time ratio (dm/dt) of 0.0005% min^−1^.

### 2.11. Solubility Test

The solubility of SAP in a release medium (0.01 M PBS, pH = 7.4) was assessed in triplicate by solvent saturation method. An excess amount of SAP was added into tubes containing 2.5 mL of PBS. The tubes were sonicated into a water-bath sonicator at 32 KHz and 32 °C, for 1 h, and then stirred on a thermostated orbital shaker at 120 rpm and 32 °C, for 24 h. The tubes were then centrifuged at 2000 rpm for 5 min. The supernatants were withdrawn, filtered using 0.22 µm polyamide syringe filters, diluted with PBS and analyzed by UV spectroscopy.

### 2.12. In Vitro Drug Release Studies 

For topical microcarriers, there are no compendial or standard release methods and apparatuses [31,32]. Therefore, in vitro release profiles of SAP from HA and HA-CL MS were evaluated with two different methodologies, under different experimental conditions.

#### 2.12.1. Dialysis

SAP release profiles were primarily obtained with dialysis method. A calculated amount of each test formulation containing ~10 mg of SAP was placed into a preconditioned dialysis bag (Slide-A-Lyzer G2, 10kDa MWCO, Thermo Fisher Scientific, Rodano, Milano, Italy), and dialyzed against 300 mL PBS (0.01 M, pH = 7.4), a release medium already described in the literature for drug release studies of dermal carriers [31,33,34]. The whole set-up was continuously stirred at 150 rpm and maintained at 32 ± 1 °C to reflect the physiological skin temperature [31]. At predefined time intervals, 1 mL of sample was withdrawn and replaced with an equal volume of warm PBS. The released SAP was quantified by UV spectroscopy. A minimum of three replicates were performed for each test formulation.

#### 2.12.2. Franz Cells

SAP release profiles from hyaluronan MS were also investigated by Franz’s cells (25 mm internal diameter, PermeGear Inc., Hellertown, PA, USA). Polyamide filter membranes 0.45 µm pore size (Sartorius Biolab Products, Goettingen, Germany) were hydrated by sonication in deionized water for 30 min, and then cut and mounted between the receiver and donor compartments of the diffusion cells. The whole diffusion cells were put in a thermostatic bath, maintained at 32 ± 1 °C. Test formulations were placed in the donor compartments—in order to have ~2 mg of SAP on the surface of the membranes —which were closed using a wax foil (Parafilm M, Bemis Company Inc., Oshkosh, WI, USA) to prevent evaporation. The receiver compartments were filled with 23 mL PBS (0.01 M, pH = 7.4) and kept under continuous magnetic stirring at 150 rpm. At selected time points, 0.5 mL of samples were withdrawn from the receptor compartment and replaced with equal volumes of warm PBS. Samples were assayed for SAP content using UV spectroscopy. A minimum of three replicates was performed for each formulation. The idea was to get preliminary indications which, if positive, will be used to support a request to the ethics committee for a human skin study.

### 2.13. Drug Release Data Analysis

All the experimental release data were fitted to a series of statistical and kinetic models to evaluate formulations performances and to elucidate their drug release mechanisms, strictly related to the properties of the polymers. This detailed mathematical modeling study was carried out to develop and characterize our novel HA CL MS, in comparison to HA MS, with a systematic approach. 

#### 2.13.1. Similarity and Difference Factors for SAP Release Profiles

For each release method used, SAP diffusion across the membranes and SAP release profiles from HA MS and HA CL MS were statistically analyzed and compared using Fit Factors described by Moore and Flanner [35], adopted by the Food and Drug Administration guidance for dissolution testing in the industry [36]. Fit factors are models widely applied by researchers [37,38,39,40] to directly compare the difference between percentage drug released per unit time between a reference and a test formulation. The difference factor (*f*_1_) and the similarity factor (*f*_2_) were calculated using Equations (6) and (7), respectively

(6) $$f_{1}=\left\{\left[\overset{n}{\underset{t=1}{\sum }}\left|R_{t}-T_{t}\right|/\overset{n}{\underset{t=1}{\sum }}R_{t}\right]\right\}\cdot 100$$

(7) $$f_{2}=50\cdot \log\left\{{\left[1+(1/n)\sum {\left(R_{t}-T_{t}\right)}^{2}\right]}^{-0.5}\cdot 100\right\}$$

where *R_t_* and *T_t_* are percentages of drug released at a certain time point (*t*) from the reference and the test formulation, respectively; *n* is the number of dissolution sampling times. The difference factor (*f*_1_) calculates the percent difference between the reference and the test curves at each time point thus measuring the relative error between the two curves. The similarity factor (*f*_2_) is a logarithmic reciprocal square root transformation of the sum of squared error and is a measurement of the similarity in percentage released between curves. For data analysis, arbitrary descriptors of difference and similarity need to be chosen: curves were considered different with *f*_1_ ≥ 10 and *f*_2_ ≤ 50. 

#### 2.13.2. Analysis of SAP Release Kinetics Using Mathematical Models

SAP release data acquired for HA and HA-CL MS were plotted into four mathematical models corresponding to the known release mechanisms. The linearized form of each function was evaluated using the R2 regression analysis, in order to understand which was the best-fit mathematical model and, therefore, the kinetic process controlling SAP release from hyaluronan MS. 

The first model used, called Zero-release kinetic, describes a release mechanism whose rate is independent of the active ingredient concentration, but it is time-dependent [41]. It is described by Equation (8)

(8) $$\frac{Q_{t}}{Q_{\infty }}=Kt+Q_{0}$$

where *Q_t_*/*Q*_∞_ is the ratio between the cumulative percentage of drug released at time *t* and at infinite time, *k* is the zero-order release constant, *t* is the time, and *Q_0_* is the initial quantity of drug in solution due to an immediate releasing process (most times *Q_0_* = 0).

On the contrary, the First order model delineates a process where the release rate is concentration dependent [41]. It is represented by Equation (9)

(9) $$log\;log\;\frac{Q_{t}}{Q_{\infty }}=log\;log\;Q_{0}-kt/2.303$$

where *Q_t_*/*Q*_∞_ is the ratio between the cumulative percentage of drug released at time *t* and at infinite time, *k* is the first-order release constant, *t* is the time, and *Q_0_* is the initial amount of drug in solution.

Also, the Higuchi model was applied [42]. It describes drug release from a matrix system whose swelling is negligible [41,43]. Therefore, the release profile is governed by the properties of the polymeric matrix and by drug solubility, and it is described by the following equation

(10) $$\frac{Q_{t}}{Q_{\infty }}=k_{H}\sqrt{t}+Q_{0}$$

where *Q_t_*/*Q*_∞_ is the ratio between the cumulative percentage of drug released at time *t* and at infinite time, *k* is the Higuchi release rate constant, *t* is the time, and *Q_0_* is the initial quantity of drug in solution due to an immediate releasing process (most times *Q_0_* = 0).

Finally, the release data were fitted to the Korsmeyer–Peppas model, which describes the drug release from swelling-controlled systems [41,43,44,45,46]. In these polymeric systems, both diffusion and dissolution occur together, and they are quite indistinguishable. Korsmeyer–Peppas proposed the following semi-empirical equation

(11) $$\frac{Q_{t}}{Q_{\infty }}=kt^{n}+Q_{0}$$

where *Q_t_*/*Q*_∞_ is the ratio between the cumulative percentage of drug released at time *t* and at infinite time, *k* is a kinetic constant related to the structural and geometric properties of the system, *t* is the time, *n* is the release exponent (connected to geometric form), and *Q_0_* is the initial amount of drug in solution. In this model, the *n* value characterizes the release mechanism: Fickian diffusion, i.e., drug diffusive process, is prevalent for *n* ≈ 0.43; Case-II transport, i.e., polymer dissolution process, for *n* ≈ 0.89; super Case-II transport for *n* > 0.89; anomalous behavior, i.e., a superposition of diffusion and dissolution, for 0.43 < *n* < 0.89.

### 2.14. Statistical Analysis

Data are presented as mean ± SD of three independent experiments (*n* = 3). Statistical analysis was performed using GraphPad Prism software version 7.0b (GraphPad, San Diego, CA, USA). The tests used were one-way (characterization of hyaluronan solutions) or two-way (characterization of MS during the pre-formulation study) analysis of variance (ANOVA), followed by Tukey post hoc analysis for multiple comparisons. Differences between results were considered statistically significant at *p* < 0.05.

## 3. Results and Discussion

### 3.1. Pre-Formulation Study: Evaluation and Optimization of Microspheres

The present work describes the production and characterization of MS using two different hyaluronans: HA-CL (test polymer) and native HA (reference polymer already employed to formulate microspheres) [10,11]. The aim was to understand if the novel HA-CL could be a promising candidate to obtain MS intended for skin application. It is well known that particles features are affected by the properties of the starting polymer solutions and by factors related to the production method [8,23,47]. In this document, we correlated MS properties to the pH and the rheological behavior of HA aqueous phases, and to the emulsification time. This systematic approach was used in order to facilitate the development of our novel formulations, considering that this was the first study to investigate the novel HA-CL as microcarrier.

**Figure 1:**
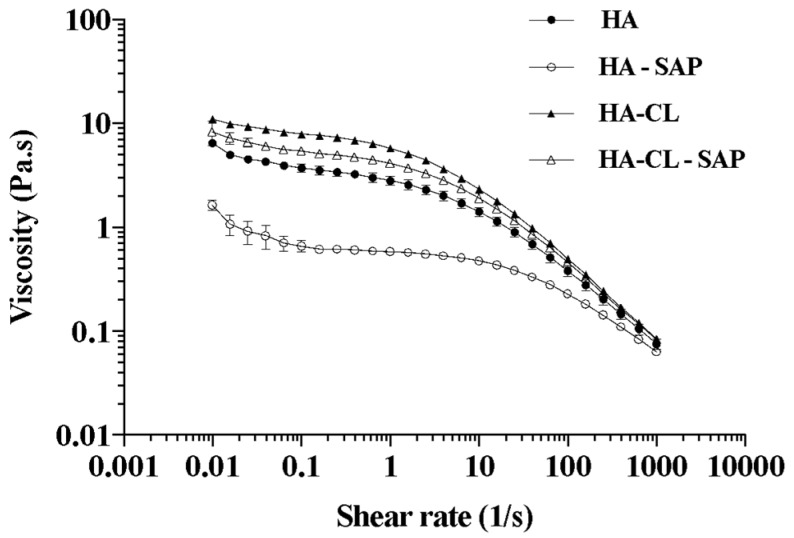
Shear-thinning behavior of hyaluronan solutions: viscosity as a function of shear rate (*n* = 3, ± SD).

All the hyaluronan solutions were characterized by a shear-thinning (Figure 1) and viscoelastic behavior. However, statistical analysis demonstrated that each solution was significantly different (*p* < 0.05) from the others in terms of pH, zero-shear-rate viscosity (*η*_0_), elastic modulus at 1 Hz (*G′_1Hz_*), and crossover frequency (Cf). Indeed, HA type and SAP presence affected the values of pH, *η*_0_, *G′_1Hz_*, and Cf (Table 1). 

As regarding the impact of polymer type, Table 1 shows implemented mechanical properties for HA-CL hydrogel with respect to HA hydrogel (i.e., higher *η*_0_ and *G′_1Hz_*, lower Cf). Considering, for example, simple polymeric solutions, *η*_0_, *G′_1Hz_* and Cf were respectively 9.1 ± 0.3 Pa.s, 13.2 ± 0.6 Pa and 1.8 ± 0.0 Hz for HA-CL, and 4.2 ± 0.2 Pa.s, 5.5 ± 0.7 Pa and 6.2 ± 1.8 Hz for HA. This could be ascribed to the different molecular weight of the two polymers (1.2 MDa for native HA, 3.0 MDa for HA-CL), and to the crosslinking of the urea derivative. Indeed, it is well known that the viscosity and the viscoelasticity of hyaluronan solutions increase with increasing polymer molecular weight [48] and crosslinking [13,49]. For the shorter emulsification times investigated (10 and 30 min), the rheological behavior of HA and HA-CL solutions seemed to be reflected in the mean size of the resulting particles. Certainly, Dv50 and D[4,3] values were significantly higher (*p* < 0.05) for HA-CL MS with respect to HA MS produced after the same mixing time (Table 2). After 10 min of emulsification, Dv50 and D[4,3] were respectively 290.0 ± 32.5 μm and 311.0 ± 32.5 μm for HA-CL MS, and 117.7 ± 20.0 μm and 246.0 ± 53.6 μm for HA MS. Moreover, after 30 min of emulsification, Dv50 and D[4,3] were specifically 181.5 ± 3.8 μm and 208.7 ± 6.7 μm for HA-CL MS, and 113.7 ± 19.6 μm and 154.7 ± 27.5 μm for HA MS. Similar findings have already been reported in the literature: bigger particles are generally produced by larger droplets of the precursory *w/o* emulsion [8], formed by increasing the solution viscosity [50].

Concerning the influence of SAP on the properties of HA and HA-CL solutions, it increased the pH, thus causing (as expected [51,52]) a statistically significant (*p* < 0.05) decrease of *η*_0_ and *G′_1Hz_*, and an increase of Cf (Table 1). In fact, the pH, *η*_0_, *G′_1Hz_* and Cf of HA hydrogel, which were respectively 6.8 ± 0.1, 4.2 ± 0.2 Pa.s, 5.5 ± 0.7 Pa and 6.2 ± 1.8 Hz, changed to 8.9 ± 0.1, 0.7 ± 0.0 Pa.s, 0.8 ± 0.0 Pa and 15.9 ± 0.4 Hz in presence of SAP. A similar trend was observed also for HA-CL solutions: without SAP, the pH, *η*_0_, *G′_1Hz_* and Cf were respectively 7.2 ± 0.0, 9.1 ± 0.3 Pa.s, 13.2 ± 0.6 Pa and 1.8 ± 0.0 Hz; in presence of SAP, these values changed to 8.1 ± 0.1, 6.3 ± 0.5 Pa.s, 9.2 ± 0.4 Pa and 3.1 ± 0.1 Hz. Also, in this case, the rheology of the initial solutions appeared correlated to the dimensional properties of the particles. Indeed, for the same HA type and emulsification time (10 or 30 min), SAP-loaded MS were smaller than unloaded MS (Table 2), as highlighted by statistical analysis (*p* < 0.05). For example, after a 10 min’ emulsification, Dv50 and D[4,3] of HA MS respectively decreased from 117.7 ± 20.0 μm and 246.0 ± 53.6 μm to 71.7 ± 4.2 μm and 121.0 ± 13.4 μm in presence of SAP. Similarly, Dv50 and D[4,3] of HA-CL MS respectively decreased from 290.0 ± 32.5 μm and 311.0 ± 32.5 μm to 245.5 ± 27.6 μm and 300.5 ± 24.7 μm in presence of SAP. In the same way, after 30 min of emulsification, HA and HA-CL MS mean size decreased when encapsulating SAP, as reported in Table 2.

In summary, the effect of HA type and SAP presence on MS diameter appeared statistically significant (*p* < 0.05) in the case of 10 and 30 min of emulsification. For MS produced after 60 min of mixing, this effect was negligible. Indeed, particles were all comparable in term of size, regardless of polymer type and SAP loading, with Dv50 ranging from 2.5 ± 0.1 to 13.0 ± 0.7 µm, and D[4,3] ranging from 3.1 ± 0.2 to 21.6 ± 4.0 µm (Table 2). Therefore, after 60 min, the side effects due to the differences between hyaluronan solutions were leveled because of a more significative emulsification. Consequently, MS mean diameter and size distribution seemed to be affected not only by the rheology of the starting solution but also by the emulsification time. For each MS formulation, particle diameter was found to be inversely proportional to the emulsification time in the range 10–60 min (Table 2). This correlation was statistically significant (*p* < 0.05), and it was in agreement with previously reported results [47]. Indeed, the higher is the mixing time the smaller are the droplets produced during the emulsification, and, consequently, the final particles. On the contrary, the shorter is the emulsification time, the less fine is the w/o emulsion, and this normally determines the aggregation of aqueous droplets. As already observed [8], also in our study bigger and less uniform-sized MS, with a lower yield, were produced by droplets agglomeration -supposed to occur especially for 10 min’ emulsification (Table 2). This drawback could be probably due to the bioadhesivity of the polymers which, when less dispersed, adhere more to the homogenizer workhead. However, by increasing the emulsification time from 10 to 60 min, MS mean size significantly decreased, while particle recovery (Y%) and encapsulation efficiency (EE%) significantly increased (*p* < 0.05) (Table 2). For example, SAP-loaded HA MS could be considered: after 10 min of emulsification, Dv50, D[4,3], Span, Y% and EE% values were respectively 71.7 ± 4.2 μm, 121.0 ± 13.4 μm, 4.3 ± 0.1, 75.2 ± 4.4% and 59.0 ± 4.0%. After 60 min of emulsification, these values became respectively 2.5 ± 0.1 μm, 3.1 ± 0.2 μm, 2.2 ± 0.0, 84.2 ± 2.3% and 69.7 ± 4.6%. Considering the parallel increment of particles Y% and EE%, the drug loading (DL%) remained almost constant whatever the emulsification time (around 40% for SAP-loaded HA MS and 33% for SAP-loaded HA-CL MS) (Table 2). 

**Figure 2:**
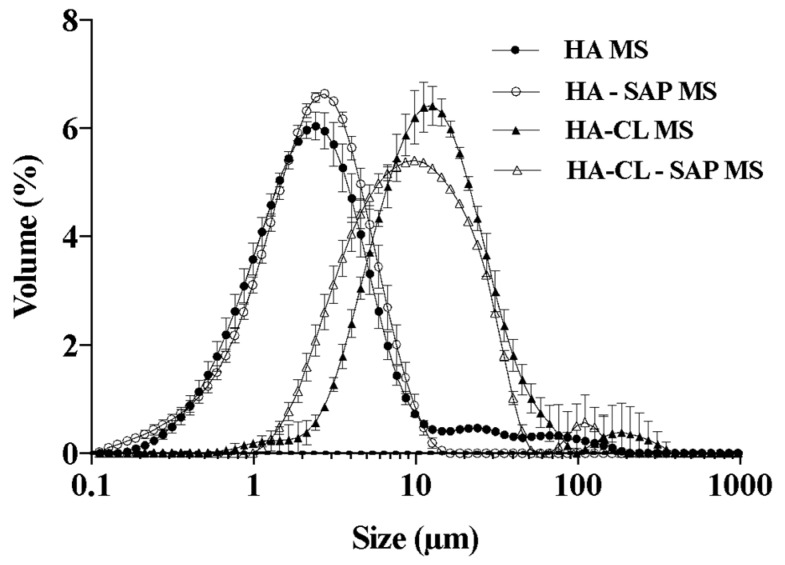
Particle size distribution of hyaluronan MS produced after 60 min of emulsification (*n* = 3, ± SD).

Taking into account the results of this pre-formulation study, the emulsification time of 60 min was chosen as the standard condition to produce optimized MS. Indeed, all the MS formulations produced after 60 min of emulsification were characterized by the highest Y%, ranging from 84.2 ± 2.3 to 88.4 ± 1.7% (comparable values). The EE% and DL% were satisfying, even if statistically different (*p* < 0.05) for HA and HA-CL formulations: respectively 69.7 ± 4.6% and 41.3 ± 1.6% for HA–SAP MS, and 78.8 ± 2.6% and 33.6 ± 2.3% for HA-CL–SAP MS (Table 2). Particle Dv50 ranging from 2.5 ± 0.1 to 13.0 ± 0.7 µm, and D[4,3] ranging from 3.1 ± 0.2 to 21.6 ± 4.0 µm (Table 2) were suitable for dermal target [53,54]. Indeed, to avoid palpable microspheres during application, mean size should be lower than 50 µm [55]: this is essential both in the cosmetic and pharmaceutic field, as it determines the cosmetic elegance of a product [55] and the adherence to a therapy [56]. A statistical comparison revealed no significant difference between the mean size of optimized MS (*p* > 0.05). Moreover, all the optimized formulations showed almost unimodal size distributions (Figure 2), with Span values lower than 3 (Table 2).

Optimized MS underwent a deeper physical–chemical characterization: analysis of morphology, physical and molecular state, thermal properties, relative moisture sorption, and stability, in vitro release properties, and kinetic mechanisms. 

### 3.2. Characterization of Optimized Microspheres

#### 3.2.1. SEM Morphological Analysis

**Figure 3:**
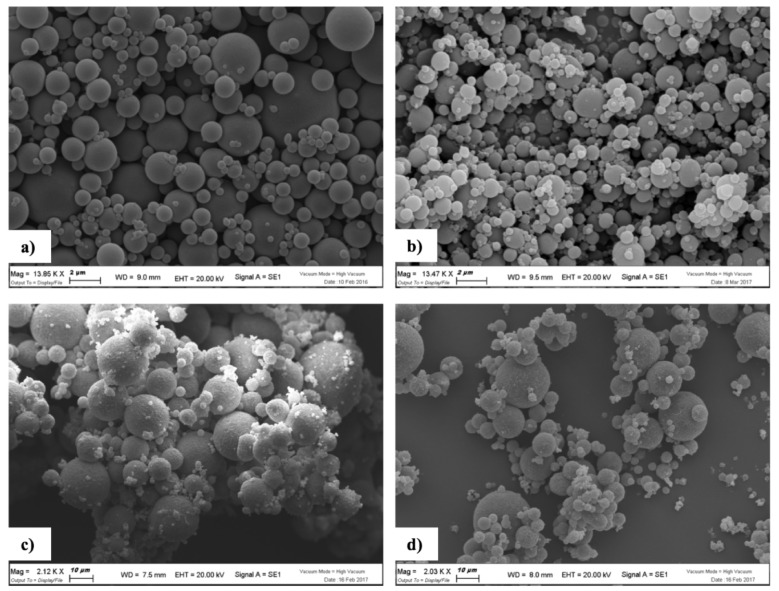
SEM micrographs of hyaluronan MS produced after 60 min of emulsification: HA (**a**), HA-SAP (**b**), HA-CL (**c**), HA-CL–SAP (**d**).

As regarding particle morphology, all hyaluronan MS encapsulating or not SAP showed a spherical shape. However, polymer typology (HA or HA-CL) influenced MS surface properties. Indeed, SAP-loaded and unloaded HA MS exhibited a regular and smooth surface, while SAP-loaded and unloaded HA-CL MS were characterized by an irregular and rough surface (Figure 3). 

#### 3.2.2. X-ray Diffraction

**Figure 4:**
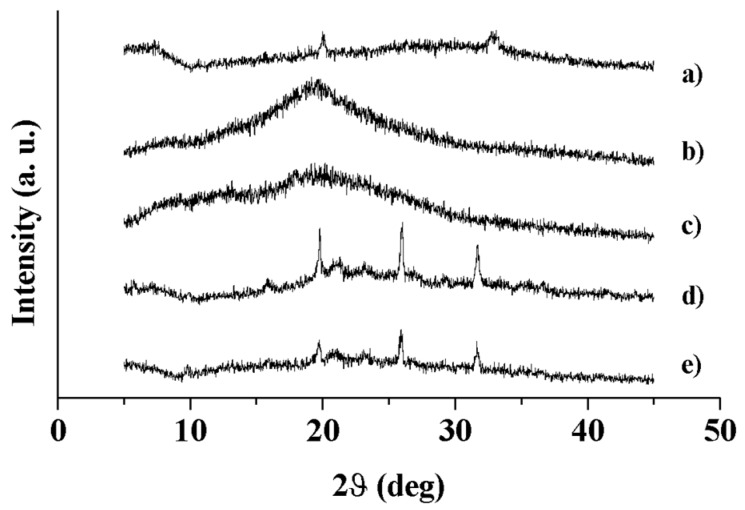
X-ray diffraction patterns of: SAP (a), HA MS (b), HA-CL MS (c), HA–SAP MS (d), HA-CL–SAP MS (e).

Wide angle X-ray diffractometry was performed to investigate the molecular states of MS formulations in comparison to SAP [57]. The diffraction patterns are reported in Figure 4. The WAXD pattern of unloaded HA and HA-CL MS exhibited humps typical of disordered structures, i.e., amorphous materials. SAP pattern was characterized by four low intense and broad peaks emerging from a hump at 2ϑ = 7.30, 20.00, 27.32, and 33.12°, which can be ascribed to small traits of crystallinity. Indeed, the diffraction intensity is defined by the crystal structure: the higher is the crystallinity degree, the higher is the intensity of the peaks [57]. The diffraction patterns of SAP-loaded MS showed both the main signals of the drug and of the carrier, indicating the permanence of SAP crystalline traits into the amorphous matrixes of HA and HA-CL. However, thermal analyses and DVS study provided evidence and confirmation that the crystallinity of SAP loaded MS was so low to be negligible.

#### 3.2.3. Thermal Analysis (DSC and TGA)

**Figure 5:**
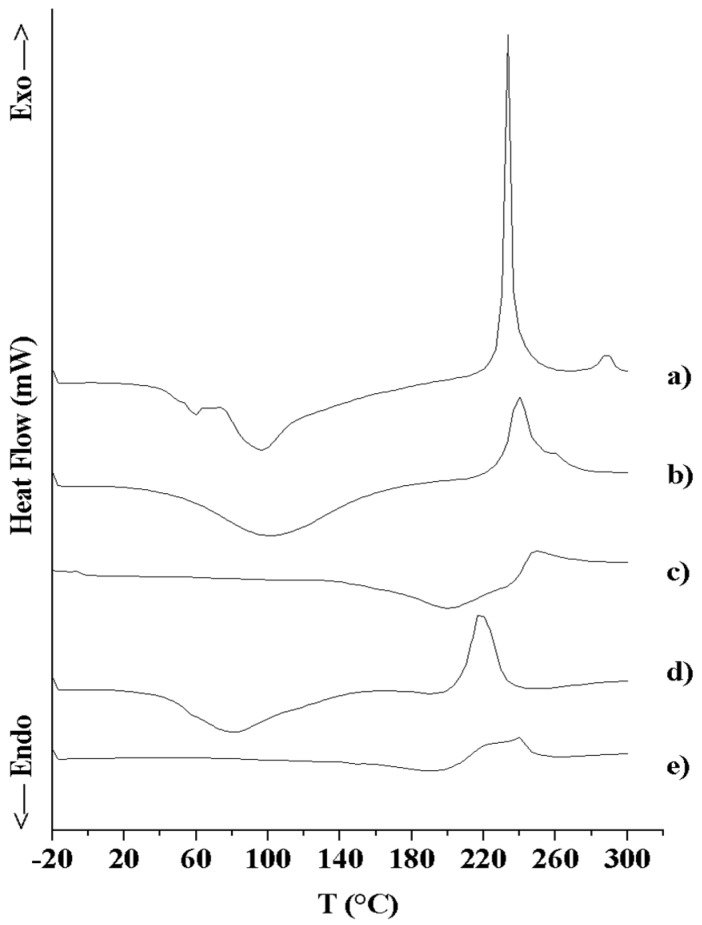
DSC thermal profiles of: SAP (a), HA MS (b), HA-CL MS (c), HA–SAP MS (d), HA-CL–SAP MS (e).

DSC and TGA were used to characterize the thermal behavior and stability of the drug and the formulations, providing information on their hydration properties and their physical state [58,59]. Figure 5 shows the DSC thermal profiles of SAP and MS formulations. SAP thermogram (a) was characterized by broad endothermic peaks around 67 °C and 100 °C, which could be associated with the loss of moisture after the initial drying procedure, and by a sharp exothermic peak at 233 °C, due to the melting point with thermal decomposition (as reported in the literature for similar ascorbic acid derivatives [60]). DSC thermal profile of HA MS (b) presented a wide endothermic peak, suggesting a dehydration process around 103 °C, and a broad exothermic peak at 240 °C ascribable to the polymer thermal decomposition and the formation of a carbonized residue. These results were in good agreement with previous observations for native HA [49,61,62]. DSC trace of HA-CL MS (c) exhibited a broad endothermic peak at 200 °C, which could be attributed to pentylene glycol evaporation (boiling range 198–200 °C). This was confirmed by pentylene glycol DSC thermal profile (trace not shown). Moreover, HA-CL MS curve showed a wide exothermic peak, due to polymer thermal degradation, at 250 °C (shifted with respect to HA MS, indicating an altered structure due to crosslinking [61]). The thermograms of HA - SAP MS (d) and HA-CL-SAP MS (e) showed the same profile of the corresponding unloaded MS (DSC traces b and d, respectively), but the peaks shifted to lower temperatures (about 10–15 °C of shift). This evidence, in addition to the reduced intensity of the exothermic peak, suggested an altered microstructure of the polymer matrix due to SAP presence (probable molecular dispersion of SAP inside the microspheres [24]) and the superposition of SAP and hyaluronan thermal degradation phenomena.

**Figure 6:**
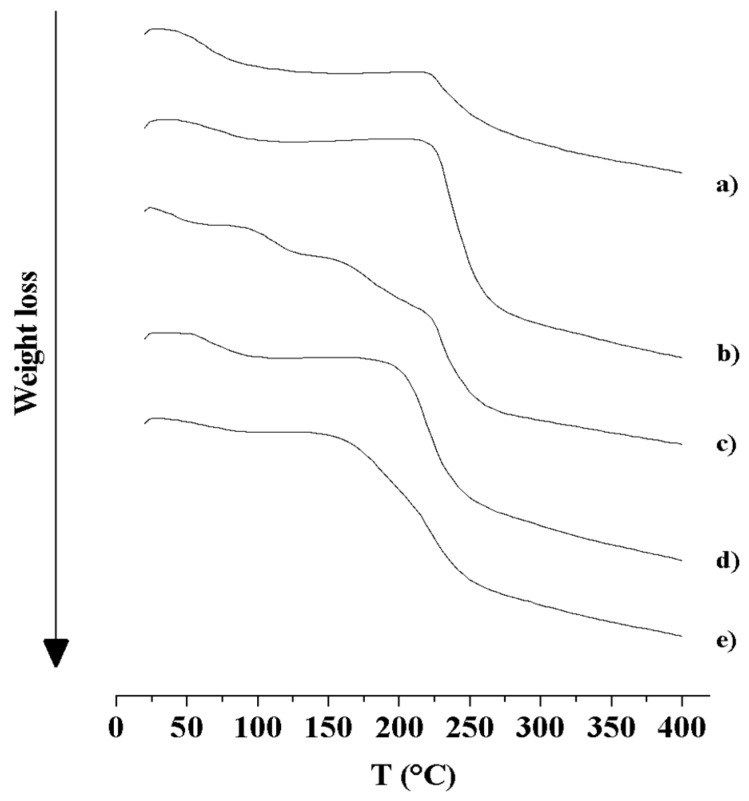
TGA thermograms of: SAP (a), HA MS (b), HA-CL MS (c), HA–SAP MS (d), HA-CL–SAP MS (e).

TGA thermal profiles of SAP and MS formulations are reported in Figure 6. SAP thermogram (a) showed the first region of weight loss (9.6% *w*/*w*) between ambient temperature and 223 °C (moisture loss), and a second region (20.5% *w*/*w*) from 223 to 400 °C (melting with decomposition and release of volatile degradation products). TGA curve of HA MS (b) consisted of three distinct degradation stages: the first one (20–223 °C, showing 6.2% *w*/*w* of weight loss, due to water evaporation); the second (223–269 °C) and the third stages (269–400 °C), typical of a two-stages polysaccharide degradation. In the second stage, the 37.3% *w*/*w* of weight was lost due to a partial breakage of the molecular structure. Residues of hyaluronan were then degraded in the third stage, characterized by the 12.0% *w*/*w* of weight loss. Similar findings have already been described in the literature for native HA [63,64,65]. A comparable TGA profile characterized HA-SAP MS (d), with 10.8% *w*/*w* of weight loss in the first region (20–208 °C), 26.2% *w*/*w* in the second (208–258 °C), and 12.0% *w*/*w* in the third (258–400 °C). For HA-SAP MS the decomposition of polymer and drug seemed to occur at once. TGA thermograms of HA-CL MS (**c**) and HA-CL-SAP MS (e) presented the two-stages polysaccharide degradation observed also for HA MS formulations (b and d), with an additive stage for pentylene glycol evaporation and more stages for water loss. The more gradual moisture evaporation was due to the water-binding action of pentylene glycol, humectant contained in HA-CL matrix. In detail, TGA trace of HA-CL MS (c) exhibited the following weight loss regions: three stages for water and then pentylene glycol evaporation (20–97 °C, 97–158 °C, 158–223 °C -total weight loss: 26.2% *w*/*w*), and two stages for HA degradation (223–265 °C, 223–400 °C -total weight loss: 29.5% *w*/*w*). TGA thermal profile of HA-CL-SAP MS (e) was characterized by two regions for moisture and then pentylene glycol loss (20–154 °C, 154–212 °C, a total weight loss of 21.5% *w*/*w*), and two regions for HA-CL and SAP decomposition (212–254 °C, 254–400 °C, a total weight loss of 30.3% *w*/*w*).

#### 3.2.4. Dynamic Vapor Sorption (DVS)

**Figure 7:**
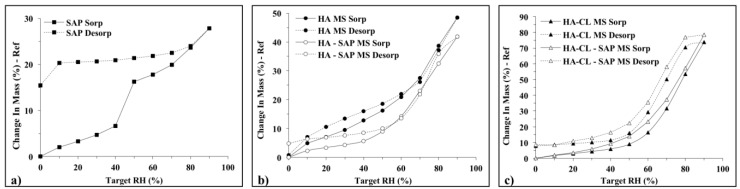
DVS isotherms of the first cycle sorption-desorption for: SAP (**a**), HA MS and HA–SAP MS (**b**), HA-CL MS and HA-CL–SAP MS (**c**).

It is well known that HA is a highly hygroscopic macromolecule, therefore by nature susceptible to moisture sorption and elevated relative humidity (RH) [6,66]. To further characterize hyaluronan MS, and evaluate the effect of SAP encapsulation, the drug, and the formulations were subjected to two 0–90% RH cycles using a DVS. The isotherms of water sorption and desorption for the first humidity ramp (cycle 1) are shown in Figure 7 as a function of the RH%. Following the sorption data of SAP (panel (a)), there was a linear increase of water content starting from the dry powder up to an RH of 40%, where the moisture uptake was 6.6%. A steeper increase of water sorption (+ 9.6%) was observed in the RH range 40–50%. The final total humidity absorbed by SAP was 27.8%. The SAP desorption curve had a very different profile from the SAP sorption curve. A pronounced hysteresis was observed, and at the end of the desorption process, the final retained moisture was 15.4%. On the one hand, this suggested that SAP was not reversible in terms of humidity sorption/desorption, and that water molecules were not easily detached from it, perhaps because of their condensation among the hydrophobic skeleton of the drug. On the other hand, MS formulations (panels (b) and (c)) showed, as previously observed for native HA [67,68], similar trends for their sorption and desorption ramps, and high water-binding capacity due to H-bonds and electrostatic interaction with hyaluronan hydroxyl and carboxylic groups, respectively. Two-stages moisture sorption processes occurred for hyaluronan MS formulations, and this was in agreement with data already reported in the literature for native HA [67,68]. Indeed, in response to RH increment from 0 to 60%, water uptake slowly increased up to 20.9% for HA MS, 14.2% for HA–SAP MS, 16.4% for HA-CL MS, 23.0% for HA-CL–SAP MS (changes in mass similar to that of SAP (+ 17.8%) at 60% RH). However, the final water retention at the end of the sorption process was higher for MS formulations with respect to SAP, as moisture uptake was markedly enhanced for all the formulations in the RH range 60–90%. At 90% RH, the water content was 48.5% for HA MS, 41.9% for HA–SAP MS, 73.8% for HA-CL MS, 78.4% for HA-CL–SAP MS. The higher moisture sorptions observed for HA-CL formulations compared to HA formulations were most likely due to the water-binding ability of urea and pentylene glycol. All the MS formulations displayed hysteresis phenomena: for the same RH value, during the desorption process, samples were characterized by a higher moisture level than during the sorption procedure. However, the hysteresis was reduced with respect to SAP, as well as the final moisture level at the end of the desorption process (0% RH), which was 0.8% for HA MS, 4.9% for HA–SAP MS, 7.8% for HA-CL MS, 8.7% for HA-CL–SAP MS. Therefore, the encapsulation of SAP into hyaluronan MS produced formulations more reversible in terms of moisture sorption/desorption compared to the pure drug, even if more susceptible to high RH%.

#### 3.2.5. SAP Solubility

The solubility of SAP in PBS (0.01 M, pH = 7.4) at 32 °C was 425.0 ± 0.9 mg/mL. This result displayed that sink conditions were guaranteed during the in vitro drug release studies, as SAP concentration, in the case of complete release, could reach values of 0.033 mg/mL in the dialysis tests, and 0.087 mg/mL in the Franz diffusion cell tests.

#### 3.2.6. In Vitro Drug Release Studies and Kinetic Analysis 

One of the most important steps in the study of the efficacy of new delivery systems is the in vitro drug release analysis. Topical carriers are an advanced form of powders for which, so far, there are no compendial or standard release techniques and apparatuses [31,32]. Therefore, several in vitro drug release methods have been used: for example, dialysis [8,12,31,69], Franz cells [23,31,40,70], paddle or basket apparatuses [11,71], flow through cells [8,40]. Variations in the release profiles between different methodologies could be observed [8,31,40], as the methods are different in their working principles. Therefore, during this study, SAP release from HA and HA-CL MS was evaluated with two different in vitro methods: dialysis and Franz diffusion cells. As the control, diffusion tests of free SAP across dialysis and polyamide filter membranes were performed.

**Figure 8:**
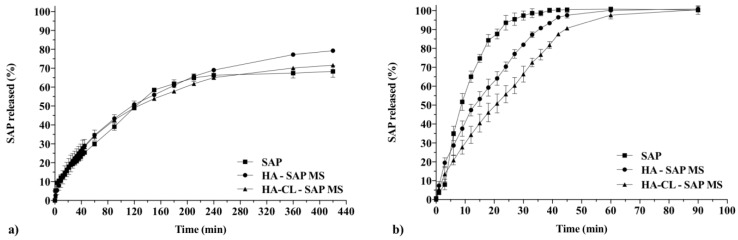
Diffusion profile of SAP as free drug and release profile of SAP from HA and HA-CL MS investigated by dialysis (**a**) and Franz diffusion cells (**b**).

Dialysis is a widely used release technique: microspheres are retained into a membrane, while the drug released diffuses firstly from the carrier to the media inside the membrane, and then to an external compartment. Inevitably, the membrane opposes a resistance to the diffusion of drug molecules. This resistance can be limited by using a membrane with an MWCO smaller than the microcarrier size, but importantly bigger than drug molecular weight [72]. Considering hyaluronan MS size and SAP molecular weight (358.08 g/mol), 10kDa MWCO dialysis cassettes were employed in this study. Nevertheless, significant errors were introduced by this release method, as the experimental data did not seem to fully reflect the real release profile of SAP from hyaluronan MS. As previously reported in the literature [72,73], the measured release kinetic seemed to be decreased with respect to the reality: the diffusion rate of free SAP, a highly hydrophilic drug, appeared extremely slow—after 420 min, only the 68.3 ± 3.1% of the drug diffused (Figure 8a). Moreover, SAP release profiles from MS appeared controlled by the membranes rather than by the microspheres, as they were almost identical to the diffusion profile of free SAP (Figure 8a). Indeed, a statistical comparison of dialysis data with Moore and Flanner Fit Factors confirmed that all the curves were similar, showing *f*_1_ < 10 and *f*_2_ > 50 (Table 3). Therefore, in this work, the precise release process and kinetic behavior could not be studied using dialysis release method. Similar drawbacks using dialysis technique have already been described in the literature for procaine hydrochloride release from a polymeric carrier: the actual rate of drug release from the delivery system was faster than the rate of diffusion out of the membrane [72]. These results confirm that dialysis could be an unreliable methodology to study drug release from microcarriers, especially when characterized by a rapid release kinetics [72,73]. Taken together, all these considerations and experimental pieces of evidence can be helpful to optimize, in future, the design of drug release studies for microsystems. 

Franz diffusion cell tests are probably the most suitable and performed studies to investigate plain drug diffusion and drug release from microparticles intended for dermal and/or mucosal application. Indeed, this model reproduces the conditions encountered on skin and mucosae surface, consenting a slow hydration of the carrier in a humid environment. In vitro release profiles obtained with Franz diffusion cells method were different from those observed with dialysis technique, as displayed by Figure 8. Free SAP diffusion was faster than SAP release from HA MS and HA-CL MS (Figure 8b). More precisely, SAP release from HA-CL MS was significantly extended not only compared to plain SAP but also with respect to HA MS, as proved by Moore and Flanner similarity and difference factors (*f*_1_ > 10 and *f*_2_ < 50) (Table 3). For example, after 30 min, the amount of drug released was 97.4 ± 2.4% for plain SAP, versus 81.9 ± 0.4% and 66.4 ± 4.2% for HA MS and HA-CL MS, respectively (Figure 8). A slower drug release was expected with HA-CL MS, considering the higher molecular weight and the implemented mechanical properties of the crosslinked polymer compared to native HA [9,16,74]. The influence of the polymer properties on the release profile was evident, and therefore Franz diffusion cell was found to be a discriminative method to study SAP release kinetic processes. 

Among the mathematical templates used to analyze SAP release kinetic from hyaluronan MS, Korsmeyer**–**Peppas model resulted in the highest *R*^2^ values when compared to Zero order, First order and Higuchi models, for both the formulations (Table 4). The values of the diffusional exponent *n* characterizing the release mechanism were 0.709 for HA–SAP MS and 0.712 for HA-CL–SAP MS, suggesting anomalous transport processes [43,46]. SAP release from hyaluronan MS was therefore governed not only by drug diffusion but also by polymer swelling and dissolution. Hyaluronan MS containing SAP behaved as swellable devices composed by hydrophilic polymeric matrixes where a water-soluble drug was dispersed: water penetrated into the polymeric networks, causing their disentanglement and swelling. These phenomena decreased hyaluronan concentration, therefore enhancing its dissolution at the interface and SAP wettability and diffusion [43,46]. Thus, SAP release was completed in a time frame of about an hour for both the formulations (Figure 8). 

## 4. Conclusions

The present study showed, for the first time and with a systematic approach, that HA-CL could be a promising biopolymer to prepare drug-loaded microspheres with a water-in-oil (w/o) emulsification solvent evaporation technique. Appropriate working conditions led to the production of HA-CL MS and HA-CL**–**SAP MS characterized by almost unimodal size distributions (*Span* values lower than 3); mean diameter of 13.0 ± 0.7 and 9.9 ± 0.8 µm, respectively (suitable for dermal application); spherical shape and rough surface; high yield—similar to that of HA MS and HA**–**SAP MS (≈ 85%). SAP could be more efficiently encapsulated into HA-CL MS (78.8 ± 2.6%) compared to HA**–**SAP MS (69.7 ± 4.6%). Physical and molecular state, thermal properties, relative moisture stability of HA-CL MS and HA-CL–SAP MS were comparable to those of HA MS and HA**–**SAP MS. However, a preliminary Franz diffusion cells test displayed a more extended drug release for HA-CL**–**SAP MS with respect to HA–SAP MS, despite the same kinetic mechanism (contemporaneous drug diffusion and polymer swelling and dissolution). This underlined that the implemented mechanical properties of the novel HA-CL could result in more efficient microsystems, which could be potentially improved, in future, by the addition of excipients able to further slow down drug release. In vitro studies on skin cells are currently performed to explore SAP release/transport across the cells and the bioactivity of HA-CL MS and HA-CL**–**SAP MS, in order to understand if they effectively improve hydration and re-epithelialization compared to HA MS and HA**–**SAP MS. 

## 5. Patent

Fallacara, A.; Vertuani, S.; Manfredini, S.; Citernesi, U.R. 2018c. Patent Appl. Filed, n. 102018000008192.

## Figures and Tables

**Table 1:** Main properties of HA solutions: pH, *η_0_*, *G*′*_1Hz_*, *C_f_* (*n* = 3, ± SD).

Solution	pH	*η_0_* (Pa.s)	*G*′*_1Hz_* (Pa)	*C_f_* (Hz)
**HA**	6.8 ± 0.1	4.2 ± 0.2	5.5 ± 0.7	6.2 ± 1.8
**HA-SAP**	8.9 ± 0.1	0.7 ± 0.0	0.8 ± 0.0	15.9 ± 0.4
**HA-CL**	7.2 ± 0.0	9.1 ± 0.3	13.2 ± 0.6	1.8 ± 0.0
**HA-CL–SAP**	8.1 ± 0.1	6.3 ± 0.5	9.2 ± 0.4	3.1 ± 0.1

**Table 2:** Effect of emulsification time, polymer type, and SAP presence on MS properties (*n* = 3, ± SD).

MS Formulation	Dv50 (μm)	D[4,3] (μm)	Span	Y(%)	DL(%)	EE(%)
*10 min*						
HA	117.7 ± 20.0	246.0 ± 53.6	4.2 ± 0.4	68.6 ± 1.6	-	-
HA–SAP	71.7 ± 4.2	121.0 ± 13.4	4.3 ± 0.1	75.2 ± 4.4	39.2 ± 0.4	59.0 ± 4.0
HA-CL	290.0 ± 32.5	311.0 ± 32.5	1.5 ± 0.2	56.7 ± 5.4	-	-
HA-CL–SAP	245.5 ± 27.6	300.5 ± 24.7	2.4 ± 0.5	70.6 ± 1.1	32.5 ± 1.4	65.3 ± 3.5
*30 min*						
HA	113.7 ± 19.6	154.7 ± 27.5	2.9 ± 0.1	79.4 ± 2.9	-	-
HA–SAP	54.6 ± 3.0	63.2 ± 2.6	2.0 ± 0.1	81.5 ± 1.4	39.3 ± 1.0	64.1 ± 2.9
HA-CL	181.5 ± 3.8	208.7 ± 6.7	2.5 ± 0.1	76.7 ± 1.3	-	-
HA-CL–SAP	117.0 ± 4.2	135.0 ± 3.5	2.3 ± 0.0	78.2 ± 3.3	32.1 ± 1.0	68.9 ± 1.0
*60 min*						
HA	2.5 ± 0.1	6.3 ± 0.5	2.9 ± 0.2	88.4 ± 1.7	-	-
HA–SAP	2.5 ± 0.1	3.1 ± 0.2	2.2 ± 0.0	84.2 ± 2.3	41.3 ± 1.6	69.7 ± 4.6
HA-CL	13.0 ± 0.7	21.6 ± 4.0	2.5 ± 0.3	85.8 ± 4.4	-	-
HA-CL–SAP	9.9 ± 0.8	15.2 ± 4.0	2.6 ± 0.2	85.0 ± 4.8	33.6 ± 2.3	78.8 ± 2.6

**Table 3:** Similarity factors (*f*_2_) and difference factors (*f*_1_) for free SAP and MS formulations

Release Method	Reference Formulation	Test Formulation	*f*_1_	*f*_2_
Dialysis	SAP	HA–SAP MS	7.9	71.4
	SAP	HA-CL–SAP MS	7.0	74.9
	HA–SAP MS	HA-CL–SAP MS	3.3	82.5
Franz diffusion cell	SAP	HA–SAP MS	15.4	42.7
	SAP	HA-CL–SAP MS	27.4	30.8
	HA–SAP MS	HA-CL–SAP MS	15.1	47.9

**Table 4:** Correlation coefficient for Zero order, First order, Higuchi, and Korsmeyer**–**Peppas models for SAP dissolution profiles obtained with Franz diffusion cell method.

Formulation	Correlation Coefficient R^2^			
	Zero Order	First Order	Higuchi	Korsmeyer-Peppas
HA–SAP MS	0.729	0.941	0.929	0.993
HA-CL–SAP MS	0.837	0.922	0.960	0.999
